# Diversity and Enzymatic Profiling of Halotolerant Micromycetes from Sebkha El Melah, a Saharan Salt Flat in Southern Tunisia

**DOI:** 10.1155/2014/439197

**Published:** 2014-07-16

**Authors:** Atef Jaouani, Mohamed Neifar, Valeria Prigione, Amani Ayari, Imed Sbissi, Sonia Ben Amor, Seifeddine Ben Tekaya, Giovanna Cristina Varese, Ameur Cherif, Maher Gtari

**Affiliations:** ^1^Laboratoire Microorganismes et Biomolécules Actives, Faculté des Sciences de Tunis, Université Tunis El Manar, Campus Universitaire, 2092 Tunis, Tunisia; ^2^Dipartimento di Scienze della Vita e Biologia dei Sistemi, Università degli Studi di Torino, Viale Mattioli 25, 10125 Torino, Italy; ^3^Laboratoire Biotechnologie et Valorisation des Bio-Géo Ressources, Institut Supérieur de Biotechnologie de Sidi Thabet, Université La Manouba, 2020 Sidi Thabet, Tunisia

## Abstract

Twenty-one moderately halotolerant fungi have been isolated from sample ashes collected from Sebkha El Melah, a Saharan salt flat located in southern Tunisia. Based on morphology and sequence inference from the internal transcribed spacer regions, 28S rRNA gene and other specific genes such as *β*-tubulin, actin, calmodulin, and glyceraldehyde-3-phosphate dehydrogenase, the isolates were found to be distributed over 15 taxa belonging to 6 genera of Ascomycetes: *Cladosporium* (*n* = 3), *Alternaria* (*n* = 4), *Aspergillus* (*n* = 3), *Penicillium* (*n* = 5), *Ulocladium* (*n* = 2), and *Engyodontium* (*n* = 2). Their tolerance to different concentrations of salt in solid and liquid media was examined. Excepting *Cladosporium cladosporioides* JA18, all isolates were considered as alkali-halotolerant since they were able to grow in media containing 10% of salt with an initial pH 10. All isolates were resistant to oxidative stresses and low temperature whereas 5 strains belonging to *Alternaria*, *Ulocladium,* and *Aspergillus* genera were able to grow at 45°C. The screening of fungal strains for sets of enzyme production, namely, cellulase (CMCase), amylase, protease, lipase, and laccase, in presence of 10% NaCl, showed a variety of extracellular hydrolytic and oxidative profiles. Protease was the most abundant enzyme produced whereas laccase producers were members of the genus *Cladosporium*.

## 1. Introduction

Sebkhas are salt flats occurring on arid coastline in North Africa, Arabia, Baja California, and Shark Bay Australia [1]. They are considered among the most poikilotopic environments and characterized by extreme salt concentrations and electromagnetic radiation exposure together with low water and nutrient availabilities [2]. Regarded as detrimental to “normal subsistence,” organisms copying such conditions to survive and thrive are designed extremophiles [3]. Beside halophytes plants and algae, the mostly diverse dwellers of sebkhas being unveiled are members of bacterial, archaeal, and fungal ranks [4–8]. Members of fungi kingdom recovered from extreme environments such as sebkhas' have shed light on two promising viewpoints: first, as model for deciphering stress adaptation mechanisms in eukaryotes [9] and secondary, as novel and largely unexplored materials for the screening of novel bioactive natural products [10]. Over the past decade, there is an increased awareness for new hydrolytic enzymes useful under nonconventional conditions [11].

Sebkha El Melah, a Saharan salt flat of southern Tunisia, has an area of approximately 150 km² and the level is slightly below the sea. Fluvial basin excavation of Sebkha El Melah appeared at the beginning of the Würmian Quaternary period [12]. Around 40,000 BP the lagoon was highly desalinated by freshwater arrivals. At the upper Würm, seawater withdrew and the basin evolves to a temporary lake or continental sebkha. More recently, around 8000 years BP, the lagoon evolved into an evaporite basin. The sebkha sediments are composed of several saliferous layers of rock salt and gypsum (calcium sulfate) and/or polyhalite (sulfate of potassium, calcium, and magnesium) [12]. Here we report the isolation of moderately halotolerant fungi from Sebkha El Melah. Strains have been identified based on morphological and molecular markers and their resistance to salt, thermal, alkaline, and oxidative stresses was assessed. Their ability to produce different hydrolytic and oxidative enzymes under salt stress was also evaluated.

## 2. Material and Methods

### 2.1. Sampling Site Description and Fungal Isolation

**Figure 1:**
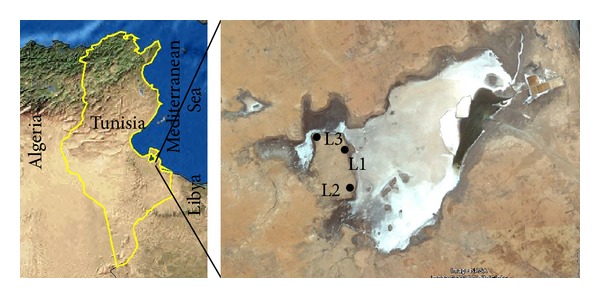
Map of Sebkha El Melah (Google Earth). L1, L2, and L3 indicate locations of sampling.

Three locations from the Sebkha El Melah margins (L1: 33°23′01.1′′N 10°54′56.8′′E; L2: 33°21′42.1′′N 10°55′05.5′′E; and L3: 33°23′37.7′′N 10°53′40.2′′E) were chosen for sampling (Figure 1). From each location, a composite sample was prepared aseptically from five subsamples (1–10 cm deep) and collected from the arms and center of an X (each arm was 1 m in length) [13]. One cm soil from the ground surface was firstly removed to avoid contamination during sampling procedure. Samples were then transported to the laboratory in a cool box and stored at 4°C prior to processing.

Fungi were isolated on potato dextrose agar (PDA) containing 10% of NaCl and 0.05% of chloramphenicol using the soil plate method where few milligrams of sample were directly spread on the agar medium. This method has a slight edge over the dilution plate method since it allows higher total number of isolates and limits invasion by species which sporulate heavily [14].

### 2.2. Morphological and Molecular Identification

Isolated fungi were identified conventionally according to their macroscopic and microscopic features. After determination of their genera [15–17], they were transferred to the media recommended of selected genus monographs for species identification.

DNA extraction was achieved as described by Liu et al. [18]; the amplification of the internal transcribed spacer regions (nuclear-encoded 18S rRNA-ITS1-5.8S rRNA-ITS2-28S rRNA) was performed using the couple of universal primers ITS1 (5′-TCC GTA GGT GAA CCT GCG G-3′) and ITS4 (5′-TCC TCC GCT TAT TGA TAT GC-3′) [19] and the thermal cycler conditions according to Luo and Mitchell [20]. PCR was carried out in 25 *μ*L volumes containing 2.5 *μ*L of 1X PCR reaction buffer (100 mM Tris-HCl, 500 mM KCl, pH 8.3), 1.5 *μ*L MgCl_2_, 0.2 *μ*mol/L (each) primer, 0.2 *μ*mol/L (each) dNTP, and 2.5 units of Taq polymerase (Dream Taq, Fermentas) and 1 *μ*L of DNA template. Depending on the fungus genus, different gene sequences were amplified. For the* Aspergillus flavus* group, the calmodulin gene was amplified using the primers CL1 (5′-GARTWCAAGGAGGCCTTCTC-3′) and CL2A (5′-TTTTGCATCATGAGTTGGAC-3′) according to Rodrigues et al. [21]; for the* Cladosporium* genus, the actin gene was amplified using the primers ACT-512F (5′-ATGTGCAAGGCCGGTTTCGC-3′) and ACT-783R (5′-TACGAGTCCTTCTGGCCCAT-3′) according to Bensch et al. [22]; for* Alternaria* genus, the glyceraldehyde-3-phosphate dehydrogenase gene was amplified using the primers GPD1 (5′-CAACGGCTTCGGTCGCATTG-3′) and GPD2 (5′-GCCAAGCAGTTGGTTGTGC-3′) according to Berbee et al. [23]; for* Penicillium* and* Aspergillus* genera, the *β*-tubulin gene was amplified using the primers Bt2a (5′-GGTAACCAAATCGGTGCTGCTTTC-3′) and Bt2B (5′-ACCCTCAGTGTAGTGACCCTTGGC-3′) according to Glass and Donaldson [24].

The PCR products were purified with QIAquick Wizard PCR purification Kit (Promega) according to the manufacturer's instructions, and the sequences were determined by cycle sequencing using the Taq Dye Deoxy Terminator Cycle Sequencing kit (Applied Biosystems, HTDS, Tunisia) and fragment separation in an ABI PrismTM 3130 DNA sequencer (Applied Biosystems, HTDS, Tunisia). The sequences obtained were compared reference sequences in the NCBI GenBank database using the BLASTN search option [25].

### 2.3. Effect of pH, Salinity, Temperature, and Oxidative Stress

PDA medium was used to study the effect of different stresses on solid media. For oxidative stresses, H_2_O_2_ or paraquat was filter sterilized and added separately to melted PDA medium previously autoclaved. Paraquat is a redox-cycling agent widely used to generate reactive oxygen species and induce oxidative stress in bacteria [26] and fungi [27]. For pH stress, PDA medium was buffered with 100 mM Glycine-NaOH to pH 10 before autoclaving. Salt stress in solid media was studied in PDA medium containing different concentrations of salts. The inoculated plates with 3 mm cylindrical mycelial plugs were then incubated at 30°C for oxidative, salt, and pH stresses and at 4°C and 45°C for thermal stresses, and radial growth was measured daily. Results were expressed as relative growth of fungal strains under different stresses as follows: (Colony diameter under stress/colony diameter without stress after 7 days incubation) × 100.

The effect of salinity in liquid medium was carried out in Biolog system, a commercially redox based test (Biolog Inc., Hayward, CA). Malt extracts (2%) containing 0%, 5%, 10%, 15%, and 20% of salt were inoculated by a suspension of spores and fragmented mycelium according to the supplier's instructions in 96-well microtiter plates. After 15 days incubation at 30°C, the numeric results were extracted using PM Data Analysis 1.3 software. The fungal growth was assimilated to the reduction of the redox indicator. The ability of the fungus to grow in the presence of salt was expressed as the ratio of kinetic curve surface under stress versus without stress.

### 2.4. Extracellular Enzymes Production Profiling

The capacity of fungal isolates to produce extracellular enzymes, namely, amylase, cellulase, protease, laccase and lipase, was assayed in the presence of 10% of NaCl. Inoculation was made by transferring 3 mm of cylindrical mycelial plugs on the corresponding media. Amylase production was assayed on PDA containing 1% soluble starch. Enzyme production is shown by the presence of clear halo when iodine was poured onto the plates. Cellulase production was tested on PDA medium containing 1% of carboxymethylcellulose. The presence of activity is reflected by a clear halo on red background after flooding the plates with 0.2% Congo red for 30 min. Protease production was revealed on skim milk agar by the appearance of a clear zone corresponding to casein hydrolysis/solubilization surrounding the microbial colony. The laccase production was detected on PDA medium containing 5 mM of 2,6 dimethoxyphenol (DMP). Oxidation of the substrate is indicated by the appearance of brown color. Lipase production was tested on PDA medium containing 10 mL/L of Tween 20 and 0.1 g/L of CaCl_2_. Positive reaction is accompanied by the presence of precipitates around the fungal colony. The enzymes production was expressed as activity ratio (PR) which corresponds to the activity diameter (halo of enzymatic reaction) divided by the colony diameter after 7 days incubation at 30°C.

### 2.5. Statistical Analysis

The data presented are the average of the results of at least three replicates with a standard error of less than 10%.

## 3. Results

### 3.1. Isolation and Identification of Halotolerant Fungi

Twenty-one fungal isolates were obtained on halophilic medium containing 10% NaCl and subjected to morphological and molecular identification. Seventeen strains were identified at genus level based on 28S rRNA gene sequences, while four were identified based on ITS regions. Final assignment was based on combination of morphological and *β*-tubulin, actin, calmodulin, and glyceraldehyde-3-phosphate dehydrogenase genes sequencing (Table 1). The 21 strains have been identified as* Cladosporium cladosporioides *(*n* = 2),* Cladosporium halotolerans *(*n* = 1),* Cladosporium sphaerospermum *(*n* = 2),* Alternaria tenuissima *(*n* = 1),* Aspergillus flavus *(*n* = 1),* Aspergillus fumigatiaffinis *(*n* = 1),* Aspergillus fumigatus *(*n* = 1),* Penicillium canescens *(*n* = 1),* Penicillium chrysogenum *(*n* = 3),* Penicillium *sp. (*n* = 1),* Alternaria alternata *(*n* = 3),* Ulocladium consortiale *(*n* = 1),* Ulocladium *sp. (*n* = 1),* Engyodontium album *(*n* = 1), and* Embellisia phragmospora *(*n* = 1) species. All the strains have been deposited at the Mycotheca Universitatis Taurinensis (MUT) in the University of Turin.

### 3.2. Salt Tolerance of Fungal Isolates

Salt tolerance of the fungal isolates was assessed on solid and liquid media for NaCl content ranging from 5 to 20%. In solid media, salt tolerance was estimated as relative growth represented by the ratio of colony diameter under salt stress to that without salt stress. As illustrated in Table 2, all the isolated strains succeeded to grow in the presence of 10% of salt. While 19 isolates remain able to grow under 15% NaCl, only 7 isolates tolerated 20% NaCl:* Penicillium chrysogenum *JA1 and JA22*, Cladosporium halotolerans* JA8*, Cladosporium sphaerospermum* JA2*, Cladosporium cladosporioides* JA18*, Aspergillus flavus* JA4, and* Engyodontium album* JA7.

When liquid cultures were used, fungal isolates seemed to become more sensitive to salt stress. Indeed, none of the strains was able to grow in the presence of 20% NaCl, whereas only 8 strains and 19 strains tolerated 15% and 10% NaCl, respectively (Table 2).

### 3.3. Alkaline, Temperature, and Oxidative Stress

Excepting* Cladosporium cladosporioides* JA18, all tested strains were able to grow at pH 10. All isolates were able to grow at 4°C while only five strains* Aspergillus fumigatus* JA10,* Aspergillus fumigatiaffinis* JA11,* Alternaria alternata* JA23,* Ulocladium consortiale* JA12, and* Ulocladium *sp. JA17 showed a significant growth at 45°C. All 21 strains tolerated oxidative stress generated by 10 mM H_2_O_2_ and 500 *μ*M paraquat (Table 3).

### 3.4. Enzymatic Profiling of Isolates

Among the 21 strains tested, 13 strains displayed at least one of the five-screened activities: protease, amylase, cellulase, lipase, and laccase, in the presence of 10% NaCl (Table 4). Protease and amylase were the most abundant activities shown by 9 and 6 strains, respectively. Four strains belonging to* Cladosporium* and* Penicillium* genera produced laccase while* Cladosporium sphaerospermum* JA2,* Aspergillus flavus* JA4, and* Engyodontium album* JA7 were able to produce lipase. Cellulase activity was detected only in* Penicillium *sp. JA15. 

## 4. Discussion

With regard to bacteria that have been well explored in southern desert region of Tunisia [28–31], data related to fungi are scarce and are limited to truffle and mycorrhiza, so far considered as real specialists of desert environments [32, 33]. To the best of our knowledge, this is the first report on the isolation and characterization of fungi from Tunisian desert and particularly from salt flat. A collection of 21 fungi isolates have been established from samples ashes collected from Sebkha El Melah. These alkalihalotolerant fungi have been assigned to 15 taxa belonging to 6 genera of Ascomycetes. Several studies showed that fungi belonging to* Cladosporium*,* Alternaria,* and* Ulocladium* genera were clearly predominant under desert and salty environments [34, 35]. These fungi have in common thick-walled and strongly melanized spores which are important for UV, radiation, and desiccation tolerance [10]. On the other hand, Molitoris et al. [36] reported that other halotolerant and halophilic fungi such as* Aspergillus* and* Cladosporium* spp. are predominant in saline desert soil of Dead Sea. Many* Aspergillus* species have been also reported to constitute dominant fungi in desert of Saudi Arabia and Libya [37, 38], and halotolerant species, including* Aspergillus *spp.,* Penicillium *spp., and* Cladosporium sphaerospermum,* have been consistently isolated from hypersaline environments around the globe [39]. In this study, contrary to many reports on hypersaline environments, no species belonging to the genera* Eurotium*,* Thrimmatostroma*,* Emericella,* and* Phaeotheca* [9] have been obtained, probably because of the initial alkaline pH of the Sebkha El Melah salt lake. Actually, the effect of pH on the fungal diversity is controversial. Misra [40] observed that fungal diversity varies with the pH while other investigators found no significant effect of pH values of water and soil habitats on fungal occurrence [41]. It is more likely that the number of the isolated fungi is directly correlated to the organic matter content of water, mud, and soil samples [42].

Beside the identification of the recovered fungal isolates from Sebkha El Melah, the second goal of the current study was the detection of some of their physiological and biochemical features. This allows understanding ecological adaptation to extreme environment and predicts some biotechnological usage. The 21 strains have been screened for tolerance to extreme NaCl concentrations, basic pH, temperature, and oxidative stress and for the production of important enzymatic activities in presence of 10% NaCl.

Excepting* Cladosporium cladosporioides* JA18, all isolates obtained in this study can be considered as moderately haloalkaliphilic fungi as deduced from their ability to grow at pH 10 and 10% of NaCl. However, the isolates were able to grow when salt was not added to their growing media. Excepting some* Wallemia ichthyophaga* the most strictly halophilic fungus [43], all other fungal strains known to date are able to grow without salt, a fact confirmed in our study. However, gradual decrease in fungal growth was observed with the increasing of salt concentration in the culture medium. Nineteen strains remain able to grow under 15% of NaCl, whereas 7 strains were able to tolerate 20% of NaCl. This result was confirmed by salt tolerance assay in liquid media as estimated by Biolog system. It is noteworthy that fungi were more sensitive to salt stress in liquid media than in solid media. This could be explained by the alteration of the osmotic gradient, forcing the fungi to expend more energy in the osmoregulatory processes, resulting in slower growth [44]. Moreover, at higher salt concentration death occurs.

Regarding the stress of pH, the capacity of the majority of isolates to growth at pH 10 implies firstly that some habitats in the salt lake may have a varying pH and secondly that fungi can tolerate a wide pH range.* Prima facie*, the overall results in solid and liquid media showed that* Penicillium chrysogenum* JA1 and JA3,* Cladosporium sphaerospermum* JA2 and JA13,* Cladosporium cladosporioides* JA18,* Aspergillus fumigatus* JA10,* Aspergillus fumigatiaffinis* JA11, and* Alternaria tenuissima* JA6 are the most alkalihalotolerant isolates in this study.

The tolerance of the strains to extreme 45°C was tested and results indicated that* Aspergillus fumigatus* JA10,* Alternaria alternata* JA23,* Ulocladium* sp. JA17, and* Aspergillus fumigatiaffinis* JA11 were able to grow. Of particular interest, the latter two strains retained 100% of the growth rate and biomass production as estimated by colony diameter. Moreover, their ability to grow at low temperature may allow them to better adapt to the big temperature fluctuation in desert environments. Additionally, exposure to substrates generating oxidative stress such as H_2_O_2_ at 10 mM and paraquat at 500 *μ*M did not alter significantly the growth of almost tested strains demonstrating their ability to tolerate oxidative stress. These findings may explain their presence in desert regions that are considered amongst the most stressful environments on Earth because of the high UV radiation, desiccation, increased salinity, low nutrient availability, seasonal and daily temperature variation, and solar irradiation [6, 10].

It has been postulated that microorganisms sharing a rich and particular extracellular enzymatic activities are common in harsh conditions characterizing their ecological habitat including high level of aridity, temperature, ionic strength, and particularly the low nutrient availability [31]. This implies the need by microorganisms for an effective utilization of each possible available organic compound [45]. Moreover, fungal isolates from hot desert were revealed to play an important role in seeds germination by breaking dormancy and increasing water uptake [46]. In the present study, the capacity of fungal isolates to produce extracellular enzymes was assayed in the presence of 10% of NaCl. Enzymes tested were the following: amylase for degradation of starch, abundant carbohydrate polymer in many plant tissues; protease for degradation of plant and animal proteins; cellulase which hydrolyses the cellulose, the main component of wood, ubiquitous substrate for fungi; and finally the laccase involved in plant material delignification and in the synthesis of the melanin and related compounds to protect fungi against radiation. Thirteen strains displayed high productions at least for one of the five-screened activities while no clear correlation of enzyme production profile with fungal systematic groups was noted. The abundance of protease activity is in line with previous data on fungal isolates from extreme environments showing high caseinase activities with little effect of salinity and temperature on enzyme production [36]. The relative limited number of isolates displaying cellulase, amylase, lipase, and laccase activities suggests that high concentration of salt may have an adverse effect on enzyme production and/or activity. Their energy was probably oriented to avoid salt stress due to 10% NaCl rather than the production of bioactive extrolites [47]. However, not detecting the enzyme is not absolute confirmation of an isolate inability to produce it. It could also mean that the enzyme was not released from the mycelium or that the medium is inadequate for its detection [48]. Laccase production in the presence of 10% of salt by the* Cladosporium* group may be of biotechnological interest, for example, in mycoremediation of high salty environments contaminated by organic pollutants.

In conclusion, fungal community described in this study was similar to those reported in inhospitable habitats characterized by limitation of nutrients, moisture deficit, and exposure to high solar radiation. Further studies are needed in order to elucidate their biogeochemical roles in such an extreme environment and to exploit their promising potential to produce new biomolecules such as enzymes and protective agents against oxidative stress.

## Figures and Tables

**Table 1:** Identification of fungal isolates.

Strain code	28S identification	ITS identification	Identification based on specific primers	Morphological identification	Final identification and accession number in NCBI
JA1	*Penicillium *	*Penicillium flavigenum* JX997105 (100%) *P. confertum* JX997081 (100%) *P. dipodomyis* JX997080 (100%) *P. commune* KC333882 (100%) *P*. *chrysogenum* KC009827 (100%) *P. griseofulvum* JQ781833 (100%)	*Penicillium chrysogenum* (*β*-tubulin)	*Penicillium chrysogenum *	*Penicillium chrysogenum *Thom *28S KF417559* *ITS KF417577 *

JA2	*Cladosporium *	*Cladosporium* sp. GU017498 (100%) *Hyalodendron* sp. AM176721 (100%) *C. sphaerospermum* AB572902 (99%) *C. cladosporioides* EF568045 (99%)	*Cladosporium sphaerospermum *(Actin)	*nd *	*Cladosporium sphaerospermum* Penzig *28S: KF417560* *ITS: KF417578 *

JA3	*Penicillium chrysogenum *	*Penicillium canescens* HQ607858(99%)	*Penicillium chrysogenum* (*β*-tubulin)	*Penicillium chrysogenum *	*Penicillium chrysogenum *Thom *28S: KF417561* *ITS: KF417579 *

JA4	*Aspergillus *	*Aspergillus aureofulgens* EF669617 (100%)	*Aspergillus flavus* (*calmodulin*)	*nd *	*Aspergillus flavus *Link *28S: KF417562* *ITS: KF417580 *

JA5	* nd *	*Penicillium desertorum* JX997039 (100%) *P*. *chrysogenum* KC009826 (99%)	*Penicillium canescens group* (*β*-tubulin and calmodulin)	*nd *	*Penicillium canescens *Sopp *ITS: KF417581 *

JA6	*Alternaria *	*Alternaria triticimaculans* JN867470 (100%) *A. tenuissima* JN867469 (100%) *A. mali* JN867468 (100%) *A. alternata* JQ690087 (100%)	*Alternaria tenuissima* (*GPD*)	*Alternaria alternata *	*Alternaria tenuissima* (Nees) Wiltshire *28S: KF417563* * ITS: KF417582 *

JA7	*nd *	*Engyodontium album* HM214540 (100%)		*Engyodontium album *	*Engyodontium album* (Limber) de Hoog *ITS: KF417583 *

JA8	*Cladosporium *	*Cladosporium cladosporioides* EF568045 (100%) *C. sphaerospermum* AM176719 (100%) *C. halotolerans* JX535318 (99%)	*Cladosporium halotolerans* (*Actin*)	*Cladosporium cladosporioides/halotolerans *	*Cladosporium halotolerans* Zalar, de Hoog, and Gunde-Cimerman 28S: *KF417564* *ITS: KF417584 *

JA9	*Embellisia/Chalastospora *	*Embellisia phragmospora* JN383493 (100%)	*Alternaria tenuissima similarity * *Alternaria arborescens * *Alternaria alternata *(*GPD*)	*Embellisia phragmospora *	*Embellisia phragmospora* (Emden) E.G. *28S: KF417565* *ITS: KF417585 *

JA10	*Aspergillus *	*Aspergillus lentulus* JN943567 (99%) *A. aff. fumigatus* JN246066 (99%) *A. fumigatiaffinis* HF545316 (99%) *A. novofumigatus* FR733874 (99%)	*Aspergillus fumigatus* (*β*-tubulin)	*Aspergillus fumigatiaffinis *	*Aspergillus fumigatus *Fresenius *28S: KF417566* *ITS: KF417586 *

JA11	*nd *	*Aspergillus aff. fumigatus* JN246066 (100%) *A*. *fumigatiaffinis* KC253955 (99%)	*Aspergillus fumigatiaffinis* (*β*-tubulin)	*nd *	*Aspergillus fumigatiaffinis *Hong, Frisvad, and Samson *ITS: KF417587 *

JA12	*Ulocladium *	*Ulocladium consortiale* JQ585682 (100%) *Alternaria radicina* HM204457 (99%)	*Ulocladium consortiale *(*GPD*)	*Ulocladium tuberculatum/consortiale *	*Ulocladium consortiale *(Thümen) E.G. Simmons *28S: KF417567* *ITS: KF417588 *

JA13	*Cladosporium *	*Cladosporium cladosporioides* JX868638 (99%) *C. sphaerospermum* HM999943 (99%)	*Cladosporium sphaerospermum *group (Actin)	*Cladosporium sphaerospermum *	*Cladosporium sphaerospermum *Penzig *28S: KF417568* *ITS: KF417589 *

JA14	*Cladosporium/Davidiella *	*Cladosporium cladosporioides* KC009539 (99%) *Davidiella tassiana* GU248332 (98%)	*nd *	*nd *	*Cladosporium cladosporioides *(Fresenius) G.A. de Vries *28S: KF417569* *ITS: KF417590 *

JA15	*Penicillium *	*Penicillium spinulosum* KC167852 (100%) *P. glabrum* KC009784 (100%)	*nd *	*Penicillium glabrum *	*Penicillium *sp. *28S: KF417570* *ITS: KF417591 *

JA17	*Ulocladium *	*Ulocladium consortiale* JQ585682 (100%) *U. chartarum* JN942881 (99%)	*nd *	*Ulocladium *sp.	*Ulocladium *sp. 28S: *KF417572* *ITS: KF417593 *

JA18	*Cladosporium *	*Cladosporium cladosporioides* HQ380770 (100%)	*nd *	*Cladosporium cladosporioides *	*Cladosporium cladosporioides *(Fresenius) G.A. de Vries *28S: KF417573* *ITS: KF417594 *

JA19	*Alternaria *	*Alternaria *sp. KC139473 (100%) *A. arborescens* JQ781762 (100%) *A. alternata* JN107734 (100%)	*Alternaria tenuissima * *Alternaria arborescens * *Alternaria alternata * (*GPD*)	*Alternaria alternata *	*Alternaria alternata *Keissler *28S: KF417574* *ITS: KF417595 *

JA20	*Alternaria *	*Alternaria brassicae* JX290150 (100%) *A. porri* HQ821479 (100%)	*Alternaria tenuissima * *Alternaria arborescens * *Alternaria alternata * (*GPD*)	*Alternaria alternata *	*Alternaria alternata *Keissler *28S: KF417575* *ITS: KF417596 *

JA22	*Penicillium *	*Penicillium chrysogenum* KC341721 (99%) *P. dipodomyicola* JX232278 (99%) *P. rubens* JX003126 (99%) *P. commune* JN676122 (99%)	*Penicillium chrysogenum* (*β*-tubulin)	*Penicillium chrysogenum *	*Penicillium chrysogenum *Thom *28S: KF417576* *ITS: KF417597 *

JA23	*nd *	*Alternaria alternata *JQ809324 (100%) *A. quercus* KC329620 (100%) *A. tenuissima* KC329619 (100%) *A. atrans* KC329618 (100%)	*Alternaria tenuissima * *Alternaria arborescens * *Alternaria alternata * (*GPD*)	*Alternaria alternata *	*Alternaria alternata *Keissler *ITS: KF417598 *

**Table 2:** Effect of salt concentration on fungal growth in solid and liquid media.

Strain code	Strain	Solid media (1)				Liquid media (2)			
		5% NaCl	10% NaCl	15% NaCl	20% NaCl	5% NaCl	10% NaCl	15% NaCl	20% NaCl
JA1	*Penicillium chrysogenum *	74	72	60	18	83	54	10	0
JA3	*Penicillium chrysogenum *	100	72	37	0	96	46	11	0
JA22	*Penicillium chrysogenum *	100	82	41	25	90	46	0	0
AJ5	*Penicillium canescens *	70	30	20	0	79	44	0	0
JA15	*Penicillium *sp.	83	70	34	0	53	18	0	0
JA8	*Cladosporium halotolerans *	80	68	32	18	30	0	0	0
JA2	*Cladosporium sphaerospermum *	76	64	34	22	47	19	11	0
JA13	*Cladosporium sphaerospermum *	100	49	25	0	81	79	12	0
JA14	*Cladosporium cladosporioides *	40	30	10	0	0	0	0	0
JA18	*Cladosporium cladosporioides *	58	40	24	8	63	61	4	0
JA4	*Aspergillus flavus *	90	80	48	26	56	16	0	0
JA10	*Aspergillus fumigatus *	100	76	35	0	100	57	11	0
JA11	*Aspergillus fumigatiaffinis *	100	46	25	0	52	30	12	0
JA19	*Alternaria alternata *	52	38	24	0	57	9	0	0
JA20	*Alternaria alternata *	60	40	0	0	37	24	0	0
JA23	*Alternaria alternata *	100	68	20	0	65	12	0	0
JA6	*Alternaria tenuissima *	100	60	22	0	80	55	17	0
JA9	*Embellisia phragmospora *	94	50	10	0	78	26	0	0
JA12	*Ulocladium consortiale *	72	28	0	0	32	10	0	0
JA17	*Ulocladium *sp.	100	70	28	0	67	20	0	0
JA7	*Engyodontium album *	56	36	14	10	43	10	0	0

(1) Relative growth on solid media after 7 days incubation = (⌀ colony under salt stress/⌀ colony without salt stress) × 100. (2) Relative growth in liquid media after 7 days incubation = (kinetic curve surface under salt stress/kinetic curve surface without salt stress) × 100.

**Table 3:** Effect of alkaline, thermal, and oxidative stresses on fungal growth.

Strain code	Strain	Alkaline stress (1)	Thermal stress (2)		Oxidative stress (3)	
		pH 10	4°C	45°C	H_2_O_2_ [10 mM]	Paraquat [500 *µ*M]
JA1	*Penicillium chrysogenum *	43	39	—	66	74
JA3	*Penicillium chrysogenum *	42	50	—	84	71
JA22	*Penicillium chrysogenum *	47	45	—	68	53
JA5	*Penicillium canescens *	26	28	—	59	63
JA15	*Penicillium *sp.	43	100	—	100	100
JA8	*Cladosporium halotolerans *	34	26	—	44	40
JA2	*Cladosporium sphaerospermum *	21	24	—	52	48
JA13	*Cladosporium sphaerospermum *	21	43	—	55	44
JA14	*Cladosporium cladosporioides *	34	38	—	20	31
JA18	*Cladosporium cladosporioides *	—	41	—	18	16
JA4	*Aspergillus flavus *	46	22	—	47	39
JA10	*Aspergillus fumigatus *	89	41	61	100	100
JA11	*Aspergillus fumigatiaffinis *	94	26	100	100	100
JA19	*Alternaria alternata *	49	35	—	69	89
JA20	*Alternaria alternata *	58	48	—	100	100
JA23	*Alternaria alternata *	100	83	40	57	52
JA6	*Alternaria tenuissima *	57	30	—	81	100
JA9	*Embellisia phragmospora *	58	67	—	100	100
JA12	*Ulocladium consortiale *	44	37	36	56	100
JA17	*Ulocladium *sp.	93	28	100	81	100
JA7	*Engyodontium album *	34	18	—	66	53

Relative growth of fungal strains under different stresses after 7 days incubation was expressed as follows: (1) (⌀ colony at pH 10/⌀ colony at pH 5) × 100; (2) (⌀ colony at 45°C or 4°C/⌀ colony at 30°C) × 100; (3) (⌀ colony with H_2_O_2_ or paraquat/⌀ colony without stress) × 100. —: not significant growth.

**Table 4:** Enzymes activities of fungal isolates in the presence of 10% NaCl.

Strain code	Strain	Protease	Amylase	Cellulase	Lipase	Laccase
JA1	*Penicillium chrysogenum *	++	+	−	−	−
JA3	*Penicillium chrysogenum *	++	−	−	−	−
JA22	*Penicillium chrysogenum *	+	+	−	−	−
AJ5	*Penicillium canescens *	−	−	−	−	+
JA15	*Penicillium *sp.	−	−	+	−	−
JA8	*Cladosporium halotolerans *	+	−	−	−	+
JA2	*Cladosporium sphaerospermum *	+++	−	−	+	+
JA13	*Cladosporium sphaerospermum *	−	+	−	−	+
JA14	*Cladosporium cladosporioides *	+	+	−	−	−
JA18	*Cladosporium cladosporioides *	−	−	−	−	−
JA4	*Aspergillus flavus *	+	−	−	+	−
JA10	*Aspergillus fumigatus *	−	−	−	−	−
JA11	*Aspergillus fumigatiaffinis *	−	−	−	−	−
JA19	*Alternaria alternata *	−	−	−	−	−
JA20	*Alternaria alternata *	−	+	−	−	−
JA23	*Alternaria alternata *	−	−	−	−	−
JA6	*Alternaria tenuissima *	+	−	−	−	−
JA9	*Embellisia phragmospora *	−	−	−	−	−
JA12	*Ulocladium consortiale *	−	−	−	−	−
JA17	*Ulocladium *sp.	−	−	−	−	−
JA7	*Engyodontium album *	+	+	−	++	−

AR: activity ratio = (⌀ activity/⌀ colony). −: no activity; +: AR < 1; ++: 1 < AR < 2; +++: 2 < AR < 3.
